# Identifying Low Value Malnutrition Care Activities for De-Implementation and Systematised, Interdisciplinary Alternatives—A Multi-Site, Nominal Group Technique Approach

**DOI:** 10.3390/nu13062063

**Published:** 2021-06-16

**Authors:** Alita Rushton, Kai Elmas, Judith Bauer, Jack J. Bell

**Affiliations:** 1 Department of Nutrition & Dietetics, The Prince Charles Hospital, Chermside, QLD 4032, Australia; alita.rushton@health.qld.gov.au (A.R.); kaielmas121@gmail.com (K.E.); 2 School of Human Movement & Nutrition Sciences, The University of Queensland, St Lucia, QLD 4067, Australia; j.bauer1@uq.edu.au; 3 Allied Health, The Prince Charles Hospital, Chermside, QLD 4032, Australia

**Keywords:** assistants, clinical governance, de-implementation, delegation, delivery of health care, implementation science, interdisciplinary, malnutrition, nutrition assessment, nutritional support

## Abstract

Malnutrition risk is identified in over one-third of inpatients; reliance on dietetics-delivered nutrition care for all “at-risk” patients is unsustainable, inefficient, and ineffective. This study aimed to identify and prioritise low-value malnutrition care activities for de-implementation and articulate systematised interdisciplinary opportunities. Nine workshops, at eight purposively sampled hospitals, were undertaken using the nominal group technique. Participants were asked “What highly individualised malnutrition care activities do you think we could replace with systematised, interdisciplinary malnutrition care?” and “What systematised, interdisciplinary opportunities do you think we should do to provide more effective and efficient nutrition care in our ward/hospital?” Sixty-three participants were provided five votes per question. The most voted de-implementation activities were low-value nutrition reviews (32); education by dietitian (28); assessments by dietitian for patients with malnutrition screening tool score of two (22); assistants duplicating malnutrition screening (19); and comprehensive, individualised nutrition assessments where unlikely to add value (15). The top voted alternative opportunities were delegated/skill shared interventions (55), delegated/skill shared education (24), abbreviated malnutrition care processes where clinically appropriate (23), delegated/skill shared supportive food/fluids (14), and mealtime assistance (13). Findings highlight opportunities to de-implement perceived low-value malnutrition care activities and replace them with systems and skill shared alternatives across hospital settings.

## 1. Introduction

Malnutrition is identified in at least one third of admitted hospital inpatients, adversely impacts patient outcomes such as length of stay and mortality, and contributes a significant financial burden to health care systems globally [1,2,3,4,5]. This problem requires a multifaceted, interdisciplinary approach to appropriately identify those with or at risk of malnutrition and deliver interventions to prevent and/or manage this harmful condition [6,7].

Traditional treatment guidelines, expert advice, and audited care practices have focused towards intensive individualised, dietitian administered nutrition care; however, this model of care has been identified to be unsustainable in a future of increasing hospital acuity, demand, and cost [6,8,9,10,11,12]. The transition to electronic records and the resultant unsustainable increase in referrals to Dietetics departments in Australia is an example of this problem; a recent study identified that 69.1% of inpatients with, or at risk of malnutrition were not receiving nutrition information and 74.8% were not receiving nutrition monitoring [13]. There is recognition that the current provision of health care is unsustainable and has called for the implementation of interdisciplinary, systematised models of nutrition care, including delegation to assistant staff (nutrition assistant, dietitian assistants, dietary aides, nutrition and dietetic technicians, dietary support workers, allied health assistants in dietetics) [6,9,13,14,15].

High value healthcare is a healthcare reform priority for the state of Queensland [14]. De-implementation of low value activities has taken interest of researchers across the globe, and de-implementation has been defined as “process of identifying and removing harmful, non-cost-effective, or ineffective, practices based on tradition and without adequate scientific support” [16,17,18,19,20]. Subsequently, recent value-based health care initiatives have responded to the calls to action for change [6,9,12,21,22,23]. Shifting to systematised and/or interdisciplinary alternatives is an important step towards improved service efficiencies, effectiveness, and patient reported experience measures [6,9,13]. To promote sustainable adoption of high value care, current nutrition care practices must be first evaluated to identify low value activities for de-implementation [16,17,18,19]. Literature surrounding low value nutrition care practices is unknown; therefore, this study aimed to identify and prioritise low-value malnutrition care practices for de-implementation and investigate systematised interdisciplinary alternatives, through applying a nominal group technique approach.

## 2. Materials and Methods

Workshops using the nominal group technique were undertaken at purposively sampled hospitals across Queensland participating in a state-wide malnutrition care knowledge translation to practice program [6,13,24]. Workshops were facilitated by a single experienced clinician/implementation expert, proactive facilitation was required to ensure participants adhered to the nominal group technique process. Workshop instructions and worksheets can be found in Appendix A. Participants were asked the questions “What highly individualised malnutrition care activities do you think we could replace with systematised, interdisciplinary malnutrition care?” and “What systematised, interdisciplinary opportunities do you think we should do to provide more effective and efficient nutrition care in our ward/hospital?”

These questions were answered in four stages: Stage one was “silent speculation”, where participants individually documented their ideas in brief phases or statements, without interacting with other participants. Stage two was “sharing speculations”, where the facilitator coordinated a round-robin feedback session where participants read aloud each of their ideas, one at a time, without any discussion, until all ideas were exhausted; ideas were written up on poster paper/white board by the facilitator. Stage three was “scoping solutions”, where the facilitator asked participants if they had any questions or comments regarding any of the items listed and hosted discussion around the ideas to ensure clarity. The participants were prompted to consider, as a group, how easy it would be to achieve and measure the idea, and how important the idea was overall. Stage four was “summarising solutions”, where participants voted, as individuals, on to prioritise ideas listed, this was done through placing stickers next to the listed idea participants wished to vote for; each participant was provided five stickers to use at their discretion on one or multiple ideas. Listed ideas were transcribed, exactly as written on poster paper, into a Microsoft Excel^®^ spreadsheet with vote tallies for each idea (by AR).

Data analysis was undertaken using a novel blended thematic analysis technique, including mixed deductive and inductive approaches [25]. Workshops responses were printed and cut into strips (codes which were deductively mapped by two authors (A.R. and K.E.) to one of the nutrition care process (NCP) steps (themes): screening, assessment, diagnosis, nutrition care planning and interventions (interventions), and monitoring and evaluation [11,26]. Where considered to apply across more than one of the themes, codes were allocated to the theme considered most relevant. Any uncertainties regarding allocation to NCP themes were resolved by a third author (JJB); following deductive mapping of codes directly to themes, categories under the themes of screening, assessment, diagnosis, monitoring, and evaluation were defined inductively by A.R. and confirmed by J.J.B. Any uncertainties regarding categories were resolved by a third author (K.E.) Participant votes were tallied for each of the themes and categories and presented using descriptive data and graphs.

## 3. Results

### 3.1. Demographics

Nine workshops were conducted across eight sites from February to November 2019; workshops duration ranged between 60 and 90 min. Demographics of the 63 participants can be found in Table 1. Although five stickers were provided to all participants, not all votes were cast; one workshop comprised five assistants who collectively chose not to complete the final nominal group technique round of casting of votes.

Dietitians accounted for the majority of the workshop participants (70%). Assistants represented 19% of participants, and directors (6%) and student dietitians (5%) had a similar representation. The majority of the participants were female (86%). Demographics showed a varied representation across age and number of years practicing (Table 1).

### 3.2. Low-Value Nutrition Care Activities for De-Implementation 

Participants identified 101 dietetic activities for de-implementation; these were mapped to the five themes of nutrition screening, assessment, diagnosis, intervention, and monitoring and evaluation (Table 2). 

**Figure 1:**
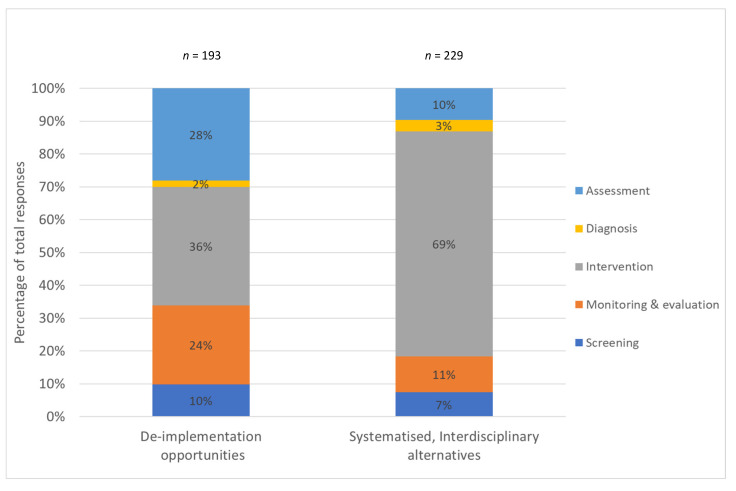
Participant vote distributions across nutrition care process steps for de-implementation actions and systematised, interdisciplinary alternatives.

Voting results demonstrated that participants were able to identify with actions across nutrition care process themes to either replace with systematised, interdisciplinary alternatives. The majority of identified disinvestment activities were spread across the Assessment, Care planning and intervention, Monitoring and Evaluation themes (Figure 1). 

#### 3.2.1. Screening Activities for De-Implementation

All votes allocated to screening de-implementation across sites were allocated to the category low value dietetics malnutrition screening, for example, *“NAs (Nutrition Assistants) and nurses doing duplicate screens on all patients”* and *“Dietitian nutrition screening in paeds wards”* (Table 3). This indicated a strong support to de-implement Nutrition Assistants’ duplicate malnutrition screening on all patients, given that all settings had clinical governance and practice processes established for nursing administered screening. The remaining votes for screening were allocated to de-implementation of screening paediatrics/low risk population groups.

#### 3.2.2. Assessment Activities for De-Implementation

De-implementation votes for the assessment theme were distributed primarily across two categories. The most popular de-implementation category was the requirement for dietitians to undertake comprehensive assessments for referrals received due to a patient scoring a positive Malnutrition Screening Tool (MST) Score of 2, for example, “*Dietitian assessment for referrals for incomplete MSTs/‘unsure’/MST 2s, and/or spending lots of time on these”* (Table 3). The other highly voted category for assessment de-implementation was dietitians and/or assistants undertaking comprehensive, individualised nutrition assessments where unlikely to add value. What this looked like across sites varied considerably, for example, *“dietitian/assistant having to chase weights/other assessment data, weekly MST, etc.”* where this is supposed to be performed by interdisciplinary team members as part of essential care, through to “*completing full assessment for patients not for enteral feeding…”* where patients were already on maximal nutrition support and documented as not for enteral tube feeding (Table 3).

#### 3.2.3. Diagnosis Activities for De-Implementation

The Diagnosis theme received the fewest de-implementation votes across all steps of the NCP. These were all allocated to “dietitian malnutrition diagnosis” (Table 3). 

#### 3.2.4. Intervention Activities for De-Implementation

The Intervention theme received the highest number of votes for de-implementation activities out of all the NCP step themes (Table 3). Votes for this theme demonstrated a broad spectrum of potential de-implementation activities that deductively aligned to the NCP intervention domains of food and nutrient delivery, education (and counselling), and coordinated care. A fourth category was also included that was considerate of multi-component intervention activities (Table 3). 

Overall, the food and nutrient delivery category accounted for 17% of votes within the intervention theme. Primarily participants considered the prescribing of Supplements as Medicine (SAM) by the dietitian as the main task which could be replaced with a systematised, interdisciplinary alternative. 

The education and counselling categories of the NCP were collapsed into one category “education” and accounted for 50% of the total de-implementation votes in the intervention theme. Almost all votes for this category were allocated to the subcategory education by dietitian to patients as an activity for de-implementation, for example, *“Dietitians doing HPHE (high protein high energy) education”* and *“Education for other,* e.g., *diverticular and gout disease”* (Table 3).

Participant responses for coordinated care were spread across several activities. The majority of the votes in this category relate to dietetics initiated coordinated care interventions considered of low value, for example *“relying on assistant/dietitian initiation of food charts*”. Another highly voted opportunity for de-implementation was dietitians providing detailed handover for low-risk patients who have been discharged from dietetics services to other service providers, for example, “*Dietitian individualised Residential Aged Care Facility (RACF) handover”* (Table 3).

#### 3.2.5. Monitoring and Evaluation Activities for De-Implementation

The most votes under the monitoring and evaluation theme were to de-implement low value review activities, for example, “*Excess reviews where unlikely to add benefit/no new action to do”*, preference checks, and dietitian reviews of oral intake (Table 3).

### 3.3. Systematised, Interdisciplinary Alternatives

When participants were asked to consider systematised, interdisciplinary alternatives, the distribution of participant identified opportunities was not evenly spread across all NCP step themes, with a disproportionate number of opportunities mapped to nutrition care interventions (Table 4). This was also reflecting in the votes cast by participants (Figure 1).

#### 3.3.1. Screening Alternatives

The majority of participants voted for opportunities to improve triaging processes associated with malnutrition screening, for example, *“MST triaging and confirmation of risk”*. Delegated or skill shared initiation of the NCP for patients at risk of malnutrition who do not require specialised care by a dietitian was also highly ranked. A *“Guilty until innocent approach for high-risk population, e.g., NOF, oncology, respiratory”,* which enabled systematised and/or interdisciplinary supportive nutrition care processes at point of identification as a high-risk patient population, without requirement for dietitian consultation, provided a key example (Table 5).

#### 3.3.2. Assessment Alternatives

The most voted systematised, interdisciplinary alternative in the assessment NCP step theme was nutrition assistants to collect nutrition assessment data, for example, “*Assistant data collection (any assessment data,* e.g., *biochemistry, anthropometry, intake, audit/monitoring)”*. The remaining votes were distributed across clinical governance matters that support improved triaging opportunities (*“using TREND [workforce planning and workload management system], using assistants, etc.—to streamline intake/prioritisation/reviews”*), assessments and re-assessments (*“Re-referral criteria, ok to discharge back to supportive nutrition care”*), and ongoing delegation of nutrition care (*“delegation/escalation/discharge criteria”)* (Table 5).

#### 3.3.3. Diagnosis Alternatives

The diagnosis theme received the lowest alternatives for current practice; however, all responses were allocated to delegated (with dietitian countersignature) or skill-shared (e.g., medical officer) diagnosis of malnutrition, for example *“Assistant facilitated SGAs [Subjective Global Assessment] (+training)”* (Table 5).

#### 3.3.4. Intervention Alternatives

The Intervention step of the NCP was the highest voted alternative (66%). Votes were again categorised to align with the NCP intervention domains (Table 5). 

Food and nutrient delivery alternatives received 12% of total intervention theme votes. Participants voted highly for delegated skill shared interventions to implement supportive food and fluid for patients rather than this being a dietitian-initiated task, for example *“Automatic nutrition supportive cart”* and *“assistant or nursing for preference checks/flavours”* (Table 5).

The education category received 21% of total intervention theme votes. Within this category, participants most frequently advocated for delegated/skill shared nutrition education, for example, *“Assistant HPHE (high protein high energy) education” and “Immediate education (e.g., nurse or assistant or doctor), e.g., using ‘you are at malnutrition risk sheet’”*. This was followed by patient engagement, for example, *“Engaging patients in care and evaluation of care (and not wasting time when not engaged”* (Table 5).

Coordination of care opportunities received 17% of total intervention theme votes. Mealtime assistance and systematised processes to support integrated care were ranked the highest within this category, for example, *“feeding assistance/mealtime assistance coordinator” and “Leveraging off Eat, Walk, Engage program”* and *“applied protocols for enteral feeds, eating disorders, etc.”* (Table 5).

The majority of votes for the intervention theme acknowledged that solutions should predominately be multi-component rather than single nutrition care processes (50% of total intervention theme). This was recognized through 55 votes for the alternative of “delegated/skill shared nutrition care processes for at risk patients who do not require specialised care for optimising malnutrition care” (Table 5). Diverse examples included *“Full scope DA [Dietitian Assistant] role”*, *“Nursing/Allied Health Assistant/Interdisciplinary [healthcare worker] to commence SIMPLE (Systematised Interdisciplinary Malnutrition Program for impLementation and Evaluation) malnutrition strategies on risk assessment”*, and *“Multi-disciplinary team or nursing assistance with supportive nutrition care at time of [malnutrition] risk screening (education, weekly weighs, intake monitoring, mealtime assistance)”*. The other key alternative was abbreviated malnutrition care processes where clinically appropriate, for example, templated documentation and reporting processes for the diagnosis, intervention, monitoring, and evaluation of malnutrition for cases where a thorough, comprehensive nutrition care process by a specialised dietitian is not indicated or likely to add value. Specific coded examples included *“Standardised chart entry template, e.g., for new/review”* and *“short malnutrition care workplace instruction and/or cognitively impaired malnutrition pathway”* (Table 5).

#### 3.3.5. Monitoring and Evaluation Alternatives

Participants most often voted for clinical governance solutions considered as opportunities to support monitoring and evaluation activities, for example, *“Clear workplace instructions, e.g., to escalate patients who are for tube feeds, and when we are not going to come back unless they are for tube feeds”*. A similar number of responses were themed to delegated or skill shared monitoring practices, for example, *“Food chart + intake review by assistant, with escalation criteria”* (Table 5). The remainder of the responses were attributable to diverse alternatives, for example malnutrition audit data. 

## 4. Discussion

To the authors knowledge, this is the first manuscript identifying clinician prioritised, low-value malnutrition care activities for de-implementation and is also the first to highlight ranked systematised, interdisciplinary alternatives considered as opportunities to provide more effective and efficient nutrition care in hospitals, highlighting many opportunities for both de-implementation and implementation. Findings demonstrate that the nominal group technique is a useful approach to prioritising embedded, low-value clinical care activities for de-implementation and systematised interdisciplinary alternatives. The individual and group elements of the framework supported establishment of consensus amongst practicing clinicians and profession management. 

These specific findings demonstrate strong consensus for enabling delegation and skill sharing activities. Our results, more broadly, however, highlight some outstanding questions that need to be considered around identified activities for de-implementation. For example, why are nutrition assistants still duplicate screening in many settings? Why do dietitians undertake comprehensive assessments with limited efficiency and effectiveness, when opportunities exist for skill-shared/interdisciplinary diagnoses? In an age where there is a clear mandate towards full or extended scope of practice [27], why are highly skilled dietitians doing low value tasks, for example, preference checks and basic nutrition education, rather than delegation or coordination of care across the care continuum? Encouragingly, however, our findings have articulated the first step; clinicians recognising the need to de-implement low value assessments, interventions and reviews. 

Despite the existence of guidelines and governance delegation to nutrition assistants and skill sharing in many settings, delegation and skill sharing of nutrition care processes are limited or do not occur at all [6,13,28,29,30,31,32,33]. Studies reporting outcomes associated with delegating or skill sharing nutrition care in healthcare settings are limited [13,34,35,36,37]. However, recent national and international nutrition care programs have demonstrated promising approaches through models of care that provide systematised, delegated, and interdisciplinary nutrition care, with evidence of sustainability and spread [13,38,39]. As an example, our study identified only a small number of votes allocated to de-implementation of dietitians diagnosing malnutrition, with alternative delegated/skill shared diagnosis, for example, by assistants or medical staff. This study is not designed to answer why it is that dietitians appear reticent to delegate or skill share malnutrition diagnosis. However, we note that delegation of malnutrition assessment to nutrition assistants is already supported by clinical governance processes; nutrition assistant completed Subjective Global Assessments (SGAs) have a state-wide authorised clinical task instruction [40]. Limited evidence also supports nutrition assistants to undertake the SGA in a rural setting [36]. We also note that due to the escalating number of positive nutrition screens associated with improved screening practices and implementation of electronic medical records, a previous study in similar settings has demonstrated that dietitians are clearly unable to deliver adequate nutrition care to the majority of inpatients screened at risk of malnutrition [6,13]. Consideration must be given to failure to ensure those screened are provided with a diagnosis and treatment in addition to the potential for case-based reimbursement disadvantage [41]. The professional impact on individual dietitians who are burdened with a high demand to undertake comprehensive nutrition assessments for a majority of cases recognised as not requiring specialised nutrition care must also be considered [13]. However, where solutions are considered that include delegated or skilled shared malnutrition diagnosis for non-complex inpatients, it is advised that this is undertaken by those who are appropriately trained, with countersignature of diagnosis by dietitians or medical officers [36,40]. 

Another de-implementation activity that has been highlighted in our results is duplicate malnutrition screening completed by nutrition assistants. According to the NCP, malnutrition screening sits outside the nutrition care process and other staff, such as nursing, are currently undertaking malnutrition screening both on admission and at defined intervals as part of workplace policies [11,42,43,44,45,46]. The results suggest that one of the practices in which efforts should be devoted for its de-implementation is lower value assistant activities, such as duplicate screening with redirection of these essential human resources to higher value opportunities for assistants that sit within the remit of the NCP [11].

As previously mentioned, 69.1% of inpatients with or at risk of malnutrition were not receiving nutrition information, and 74.8% were not receiving nutrition monitoring [13]. This being the case, it is worth reflecting on whether dietitians should be focusing practice change towards progression of delegation and skill sharing models of care for elements of assessment, diagnosis, and monitoring and evaluation steps of the NCP. 

While opportunities for de-implementation were reasonably distributed across the NCP, suggestions regarding implementation of systematised, interdisciplinary alternatives were heavily focused on the intervention step. Malnutrition assessments, diagnoses and monitoring and evaluations alternatives were not commonly identified or voted on by participants, suggesting a preference for these to remain with the dietitian, albeit with processes and guidelines to streamline/shorten the course of action required. 

We are unclear as to whether this relates to, for example dietitians wanting to retain control of these elements of the NCP. Alternatively, the results might represent distrust in systematised processes and/or interdisciplinary team members to deliver supportive but not specialised, nutrition care, noting that this is likely to be counter-productive for many patients [6,13,23]. Further speculation may indicate that the need for improved assessment/re-assessment, diagnosis, and monitoring and evaluation processes across the continuum of care is largely unrecognised by the profession. However, participant responses may have been limited to suggestions aligning with tradition nutrition care processes as a result of high-value healthcare and systematised, interdisciplinary alternatives being recently emerging concepts [6,13,14,15,21]. There is emerging evidence demonstrating the need for dietitians to de-implement low-value activities to enable opportunities to reinvest their skills to more specialised tasks [13]. 

Whether implementation and de-implementation are the flip side of the same coin is worthy of speculation [16,17,18]. In the absence of additional resources, de-implementation will always be required to resource higher value alternatives. De-implementation of low-value activities and implementation of evidence-based care is known to be influenced by several factors, such as department priorities, economic, political, and organisational factors [18,20]. Several responses in this study were related to local clinical governance structures and protocol adaptations which suggests these findings will require change and involvement from not only ward clinicians but managers and hospital/ward-based policy makers. Consideration of local barriers and facilitators to de-implementing identified low value actions and implementing the suggested alternatives are vital when applying these findings into practice. Embedding and sustaining these changes in complex healthcare settings will require careful attention to knowledge translation, implementation, trust and habit modification [22,47,48,49,50,51,52,53,54]. 

Translation into practice can be effective through applying principles of theoretical frameworks such as the “Framework describing themes regarding making change to nutrition care in hospital settings” and the “Sustain and spread framework”, both by Laur et al. [51,55]. Implementing, sustaining, and spreading nutrition care change consequently effects a change culture [51]. The culture surrounding nutrition care is crucial when considering progression of nutrition care practices and changing nutrition care processes [55]. Organizational, staff, and patient-family level practices are indicated when changing the culture of nutrition care, and building strong relationships within the hospital team is also necessary throughout the *whole* process of making changes to nutrition care in the hospital setting [51,55]. Encouraging and facilitating consideration of local factors across all practice levels and broader influential elements to change nutrition care practice may achieve longer-term nutrition care changes and healthcare cultural shifts.

A limitation of this study is that the identification of low value activities and systematised interdisciplinary alternatives were by dietetic professionals only; healthcare leaders and broader hospital ward team members were not involved in this workshop. Categories could not be deductively mapped to all individual categories of the NCP steps (e.g., assessment step categories of food/nutrition related history, anthropometric measurements, biochemical data, tests, procedures, nutrition-focused physical findings, client history), as this was not feasible based on the responses provided in the workshops being too broad. Moreover, the dietitian assistant workforce is poorly represented due to lack of attendance from nutrition assistants in the workshops. However, this study represents regional and metropolitan hospitals, with varied resources and workforces at each site. The workshops were facilitated by an experienced researcher and based on a theoretical approach (the nominal group technique) [24,56,57]. Our research appears to raise more questions than answers. Are dietitians holding on to low value activities? Are delegation opportunities, systematised models of care, and skill share activities not being clearly articulated? Is there a gap in knowledge and understanding of translating, embedding, sustaining nutrition care practice improvements [38,39]? Is there a general lack of understanding of value-based health care? Some of these questions will be explored through upcoming qualitative work in the field.

## 5. Conclusions

It is well known that malnutrition affects one in three hospital inpatients and significantly contributes to healthcare burden, and it is known that high-value healthcare is vital; this warrants reflection upon what will happen if we do not “stop” low-value malnutrition care activities, and “start” systematised interdisciplinary alternatives. Exploring and evaluating local malnutrition care practices will be useful to identify current practice of low-value activities and highlight gaps in high-value, evidenced-based practices. It is urgent that the dietetic profession responds to healthcare reform to challenge historical practice and pioneers provision of high-value healthcare by facilitating efficient, effective, and value-based nutrition care through de-implementation of low-value activities and implementation of systems, delegation, and skill sharing into routine practice.

## Figures and Tables

**Table 1:** Participant demographics.

Demographic/Variable	*n* (%)
**Position/role**	
Dietitian	44 (70)
Assistant	12 (19)
Director	4 (6)
Student dietitians	3 (5)
Gender *	
Male	7 (14)
Female	42 (86)
Age group **	
<30	11 (26)
30–39	13 (31)
40–49	11 (26)
50–59	5 (12)
60+	2 (5)
Employment contract **	
Full time	34 (81)
Part time	8 (19)
Casual	0 (0)
Number of years practicing ***	
<2 yrs	3 (6)
2–5 yrs	16 (33)
6–10 yrs	13 (27)
11–20 yrs	9 (19)
21–30 yrs	6 (13)
31+ yrs	1 (2)

* 1 site did not complete this question; ** 2 sites did note complete this question; *** 1 site did not complete this question, and 1 participant from another site did not complete this question.

**Table 2:** Identified activities for de-implementation mapped to the nutrition care process steps.

NCP Step (Theme)	*n* (%)
Screening	5 (5)
Assessment	31 (31)
Diagnosis	2 (2)
Care planning and intervention	45 (44)
Monitoring and evaluation	18 (18)

**Table 3:** Dietetics activities for de-implementation *.

NCP Step Theme and Categories	Votes *n* (%)
**Screening**	**19**
Low value dietetics malnutrition screening	19 (100)
**Assessment**	**54**
Patients with malnutrition screen score 2 by dietitian	22 (41)
Comprehensive, individualised nutrition assessments where unlikely to add value	15 (27)
Dietitian assessment prior to delegation	4 (7)
**Diagnosis**	**5**
Dietitian malnutrition diagnosis	5 (100)
**Care planning and intervention**	**58**
**Intervention**—*Food and nutrient delivery*	
Supplements As Medicine (SAM) by dietitian	5 (7)
**Intervention**—*education*	
Education by dietitian to patients	28 (41)
**Intervention**—*coordinated care*	
Low value dietitian intervention—coordination of care	5 (7)
Dietitian discharge handover low risk patient	4 (6)
**Intervention**—*multicomponent*	
Low value dietitian intervention [broad]	8 (12)
**Monitoring and evaluation**	
Low value reviews	32 (70)
Preference checks	11 (16)
Intake reviews by dietitian	10 (21)

* Only activities that received more than three votes were included in this table; however, the total NCP step (theme) votes were inclusive of all activities that received votes. Category percentages are expressed as a percentage of total theme votes.

**Table 4:** Identified opportunities for systematised, interdisciplinary alternatives mapped to the nutrition care process steps.

NCP Step (Theme)	*n* (%)
Screening	8 (7)
Assessment	11 (10)
Diagnosis	4 (4)
Care planning and intervention	75 (66)
Monitoring and evaluation	15 (13)

**Table 5:** Systematised interdisciplinary alternatives *.

NCP Step Theme and Categories	Votes *n* (%)
**Screening**	**17**
Triaging processes	9 (53)
Delegated/skill shared nutrition care process for at risk patients who do not require specialized care	4 (24)
**Assessment**	**22**
Assistant assessment data	10 (45)
Clinical governance—triaging	7 (32)
Clinical governance—assessment	4 (18)
**Diagnosis**	**8**
Delegated/skill shared diagnosis	8 (100)
**Care planning and intervention**	**157**
**Intervention**—*Food and nutrient delivery*	
Delegated/skill shared supportive food/fluids	14 (9)
Food service system	5 (3)
**Intervention**—*education*	
Delegated/skill shared education	24 (15)
Patient engagement	5 (3)
**Intervention**—*coordinated care*	
Mealtime assistance	13 (8)
Systematised processes to support integrated care	12 (8)
**Intervention**—*multicomponent*	
Delegated/skill shared nutrition care processes for at risk patients who do not require specialized care	55 (35)
Abbreviated malnutrition care processes where clinically appropriate	23 (15)
**Monitoring and evaluation**	
Clinical governance—monitoring and evaluation	11 (44)
Delegated/skill shared monitoring	10 (40)

* Only activities that received more than three votes were included in this table; however, the total NCP step (theme) votes were inclusive of *all* activities that received votes. Category percentages are expressed as a percentage of total theme votes.

## Data Availability

Please contact the corresponding author regarding data availability.

## Appendix A

**Scoping SIMPLE Solutions Workshop**

It is suggested to undertake this activity using the Nominal Group Technique.

Preparation:

1. Identify a facilitator.
2. Organise room and supplies as per the CDC guideline:
  - Pre-distributed SIMPLE infographic, summary, and pathway.
  - Local data for presentation (delegation survey graphs and dietetic survey results).
    - Current practice (DTN individually delivering, AHA individually delivering);
    - Time to provide individualised malnutrition care for ALL malnourished/at risk of malnutrition + other tasks (Q7 on DTN delegation survey);
    - Full scope perception.
  - Attendance sheet (demographics of attendees).
  - Q1 and Q2 answer sheets for step 1.
  - Large butchers paper flip chart: 3 pages (food and nutrient, education, coordination of care).
  - Markers.
  - Dot stickers for step 4 (5 per person).
  - Food/drinks.

**Welcome and introductions**

**Why the meeting is being held**

As you know, we are looking at changing the way we are managing malnutrition in [insert location]. Data from other hospitals in Queensland have shown that replacing highly individualised, dietitian focussed malnutrition care activities with systems based, interdisciplinary malnutrition care provides more effective and efficient patient focussed care.

The local data we have collected to date also show [summarise any local data that show your team should consider changing to SIMPLE malnutrition care]

**You have been invited today for 2 main reasons:**

1. Because we think you can help us to identify better ways to improve malnutrition care provided patients in hospital, every day; and
2. SIMPLE implementation will impact on your day-to-day activities.

We are also doing other activities in other areas of the hospital with other people to make sure we are engaging all the right people in this process.

**What you should have done by today**

You should have had opportunity to fill out a survey regarding malnutrition care. You should also have previously been provided with a copy of the SIMPLE Infographic, SIMPLE Summary, and SIMPLE Pathway and Opportunities. Has anybody not seen these?

**The 2 questions we want answered today are:**

1. What highly individualised malnutrition care activities do you think we could replace with SIMPLE malnutrition care?
2. What SIMPLE opportunities do you think we should do instead to provide more effective and efficient nutrition care in our ward/hospital?

We will do this in 4 stages:

**Step 1: Silently speculating**

I will present you with a question in written form and then read it to you. I will then ask you to write down your ideas in brief phrases or statements on. This is to be done on your own, without interacting with anybody else. You should record your answers on the provided question sheet.

**Step 2: Sharing speculations**

I will then coordinate a round-robin feedback session. One at a time, I will ask you to tell me each concise idea that I will write on the butchers’ paper flip chart. There will be no discussion at this time.

We will continue the round robin process until all ideas have been exhausted. Please do not repeat ideas, although feel free to raise any that have different tangents or variants.

**Step 3: Scoping solutions**

In this phase I will firstly ask “Are there any questions or comments group members would like to make about the item?” The creator of the idea does not have to be the one to answer this.

I will then host discussion around each recorded idea to ensure clarity.

Then, I will ask you to consider how easy it will be to achieve and measure, and overall, how important you think the idea is as a group (not important, somewhat important, mostly important, very important).

**Step 4: Summarising solutions**

This is where you get to vote as individuals to prioritise the ideas. Each of you will be provided with 5 round stickers; these are your voting cards. Place one sticker on each of your favourite 5 solutions; you can only vote once for each solution.

I will then tally the votes to identify which solutions your group thinks are the highest-ranking SIMPLE opportunities to implement into practice. However, remember there is still more stakeholder consultation happening, so these may not be the final outcome.

Adapted from:

https://www.cdc.gov/healthyyouth/evaluation/pdf/brief7.pdf

**Question A1:** What highly individualised malnutrition care activities do you think we could replace with SIMPLE malnutrition care? Record you answers here:

**Question A2:** What SIMPLE opportunities do you think we should do instead to provide more effective and efficient nutrition care in our ward/hospital? Record you answers here:

## Appendix B

Appendix B shows participants verbatim listed actions for de-implementation

- 1 h ax of MST 2.
- All outpatients without a screening/triaging process.
- Assistant staff “solo” meal rounds.
- “At risk” staying in “supporting”.
- Biochem data (scaffolded) by DNs.
- Blanket referrals (dependent on area -> consider locally where likely not adding value).
- Blanket referrals to Dietitian (vs, e.g., system or interdisciplinary).
- BMIs >40 individualised DN.
- Completing full assessments for pts not for enteral feeding (RACF, chronic disease, etc., frequent flyers, PEG feeds).
- Day 1 enteral tube feeding r/v.
- Detailed handovers by DNs where unlikely to add value.
- Diet restrictions.
- Dietitian (re)educations HPHE by DNs, d/c educations by DNs, f/u wound educations.
- Dietitian ax for referrals for incomplete MSTs/unsures/2s and/or spending lots of time on these.
- Dietitian SGAs/diagnosis (malnutrition).
- Director from doing HENs a/c, DNs doing HENs registration.
- Discharge referral process (e.g., PACs) for pts at risk of malnutrition/or malnourished who? Requires specialised nutrition care.
- Discharge summaries.
- Diverticulitis or HPHE counselling or other basic educations.
- DN basic malnutrition educations (but ensuring DN gives diagnosis).
- DN doing all of assessment/diagnostic, e.g., SGAs.
- DN HPHE education.
- DN individualised RACF handover.
- DN must see (at risk) for all, e.g., Pus, etc.
- DN nutrition screening in paediatrics wards.
- DN r/vs (or news for known or non-compliant) that might not change outcomes substantially.
- DN requirement for diet changes (e.g., manually putting on HPHE by DN).
- DN solo scripting.
- DN specific mealtime support.
- DN/NA having to chase weights/other ax data, weekly MST et.
- DNs and NA ongoing full r/v for “not for tube feeding”.
- DNs chasing up weights/weighing patients.
- DNs doing Ax of low risk/MST 2s, e.g., malnutrition management, oral nutrition support, educations, food charts, med pass, etc.
- DNs doing HPHE education.
- DNs doing reviews where low value unless escalated nutrition support is requested.
- DNs doing supply and distribution.
- DNs required for HP diets, etc.
- DNs spending + time on PFM triaging/new referrals, etc. for day/screening patients to be delegated.
- DN prescribing SAM.
- (Excessively) long chart entries/documentation/assessment.
- Education for other, e.g., diverticular and gout disease.
- Education of patients where does not require specialist DN education skills.
- EN/PN DN required to start.
- Excess reviews where unlikely to add benefit/no new action to do.
- Extended individual inpatient malnutrition care (e.g., 45+ min).
- Feeling bad if chart r/v not perfect.
- Flavour change/preference checks.
- Food chart and intake r/vs.
- Food intake monitoring/record charts.
- Food/flavour preferences by Dietitian.
- Foodservice complaints or issues by DNs.
- Full ax and intervention for frequent flyers.
- General HPHE educations.
- Going to [site name removed for confidentiality].
- Highly individualised enteral feed (r/vs) by DNs.
- HPHE education by DNs on low risk.
- Inappropriate referrals through PFM.
- Individualised DN care when this might be achieved using protocols or pathways or asking for DN before systems in place and applied.
- Intake and weight ax data by dietitians for non-specialised patients.
- Interventions where the outcomes does not matter to pt or is unlikely to influence outcomes.
- Lifestyle education on discharge.
- Long stay stable pts (regardless of nutrition status), e.g., palliative care.
- Low value d/c coordination for “at risk” but not specialised.
- Low value reviews by DN.
- MDT education (e.g., doctors) re malnutrition status.
- Medical round.
- Missing care (across care process) to some of the at risk.
- Monitoring setup/meal assistance.
- Monthly MST audit.
- MST “unsures”.
- MST 2 being seen by dietitian.
- MST 2 care by dietitians.
- MST 2 DN ax.
- MST 2 patients.
- MST screening by DAs.
- Multiple professions/other screening, e.g., nurses, assistants, etc.
- NA MSTs.
- NA preference checks.
- NAFLD in OPDs.
- NAs and nurses doing duplicate screens on all pts.
- Nursing home d/c reports.
- Ongoing r/vs while waiting for change, e.g., diet upgrade to the upgrade progression to tube feeds.
- Oral nutrition supplement reviews.
- Other educations by DNs on low risk.
- Over-reviews without purpose by DNs.
- Preference checks by DNs.
- Pt preferences/tolerances by DNs.
- Pump education.
- R/Vs for anthropometry/see above.
- Rechecking biochem for at risk of refeeding.
- Referrals for inappropriate weight gain.
- Reliance on D/N to prescribe SAM.
- Reliance on DN initiated MEDPASS/SAM.
- Relying on AHA/DN initiation of food charts.
- Reviewing and triaging MST scores.
- Shifting long r/v to supportive care (stop multiple individual dietetic reviews instead do supportive).
- Supplements trolley being done by a DA.
- Support reviews/preference checks by DNs.
- Thickened fluid education.
- Waiting for education by DN for MST 2+.
- Weighing patients.

Acronyms used:

Ax = Assessment
d/c = Discharge
DA = Dietitian assistant
DN = Dietitian
EN = Enteral nutrition
f/u = Follow-up
HENS = Home enteral nutrition support
HPHE = High protein high energy
MDT = Multi-disciplinary team
MST = Malnutrition screening tool
MST 2 = Malnutrition screening tool score of 2
NA = Nutrition Assistant
NAFLD = Non-alcoholic fatty liver disease
OPD = Outpatient department
PACS = Post-acute care service
PEG = Percutaneous endoscopic gastrostomy
PFM = Patient flow manager
PN = Parenteral nutrition
Pt = Patient
PU = Pressure ulcer
r/v = Review
RACF = Residential aged care facility
SAM = Supplements as medicine
SGA = Subjective global assessment

## Appendix C

Appendix C shows participants verbatim listed systematised, interdisciplinary alternative opportunities

- AHA [intake and weight ax data for non-specialised patients] with intervention plan.
- AHA audit of what care people are getting.
- AHA educations.
- AHA ensuring all at risk are getting a food and nutrition treatment.
- AHA follow-up post discharge (although may have funding issues).
- AHA HENS orders.
- AHA malnutrition education for home.
- AHA mealtime audits.
- AHA systematised HP for at risk, e.g., 7+ days.
- AHAs to see MST 2s.
- “Allowing” other HPs to provide an intervention.
- Applied protocols for enteral feeds, eating disorders, etc.
- Appropriate screening including blanket referral.
- Assistant data collection (any ax data, e.g., biochem, anthropometry, intake, audit/monitoring).
- Assistant facilitated—SGAs (+training).
- Assisted mealtimes.
- Assisted SGAs.
- Auto nutrition support cart.
- Automated d/c summary for RACF pts.
- Automated process/referral for specialised care (multiple criteria required).
- Better feeding assistance.
- Better handover, e.g., to GPs for at risk.
- Better identification of “at risk” pts (e.g., red folder/electronic/etc.)—including what is in place/could be done by team.
- Better screening beyond just MST, e.g., for ICU, #NOF, etc.
- Blanket “HP” for at risk.
- Blanket interventions [by IDT], e.g., HP or whatever, rather than waiting for DN under blanket DN.
- Clear, e.g., WPI to escalate pts who are for tube feeds, and when we are not going to come back unless they are for tube feeds.
- Clearer expectations for who could receive “supportive” vs specialist (DN) care + better referral criteria.
- DA (+ NA if can get time)—re-screening high risk (e.g., on PFM) + 7-day re-screen + repeat MST.
- DA/NA basic education.
- DA/NA chase assessment data.
- DA/NA home follow up/see if want a referral.
- DA/NA NEMO sheet and d/c planning.
- DA/NA place on HP +/- food chart.
- DA/medical/nursing/students full scope SIMPLE for MST 2/at risk (data collection, e.g., weighing, chart audit, diagnosis—PG-SGA/SGA, data collection and assisted diagnosis, HPHE education, auditing of interventions, organising food/nutrition and supplements, systematised. Not call to dietitian).
- Decrease inpatient time to allow out of hospital care or other higher value activities.
- Default HPHE (SAM) for at risk.
- Delegating or automatically generated d/c summaries.
- Delegation/escalation/discharge criteria.
- Delegation at risk direct to DAs/delegation from DN to DAs (re-escalation).
- DN and AHA team support processes (MDT meeting, board rounds, ward rounds, case conferences).
- Education + training—inservices (including local data feedback, e.g., SIMPLE audits), for interdisciplinary teams/DAs.
- Education by MDT, including d/c planning (Nursing, AHA, medical, students, other Allied Health).
- Engaging pts in care and evaluation of care (+ not wasting time when not engaged)
- Expand nutrition cart.
- External facilitator and NA together [RACF handover].
- Feeding assistance/mealtime assistance coordinator.
- Food chart + intake r/v by NA with escalation criteria.
- Foodservice staff doing food preferences/menu choices and complaints.
- Full scope DA role.
- Group based nutrition interventions with MDT like group morning teas.
- Group education for malnutrition (and/or student led).
- Guilty until innocent approach for high-risk population, e.g., NOF, oncology, respiratory.
- HENS.
- HPHE educations SIMPLE messages by IDT/AHA.
- IDT/AHA? To order drinks/supplements and other, e.g., HP diets.
- Immediate education (e.g., nurse or NA or Doctor) e.g using you are at malnutrition risk sheet.
- Imprest system/or selective mid-meal trolley/medpass.
- Initial assessment [ADIME on first time seeing pt] template for “at risk” patients (i.e., short case).
- Initial adult risk ax = add in refused DN, seen by DN in community.
- Interdisciplinary mealtime assistance and/or champions (AHA/nurses/other).
- Interdisciplinary prescription with clear guidelines.
- Interdisciplinary SIMPLE malnutrition messaging.
- It’s ok not to keep individually seeing this pt WPI.
- “It’s ok not to r/v this pt if not for tube feeding” WPI.
- “It’s ok not to see this pt anymore unless you tube feed them” standardised chart entry (i.e., we are doing everything already).
- Leverage off eat/walk/engage.
- Leverage off student workforce for SIMPLE orange.
- Local interdisciplinary or delegated process to let DN know.
- Malnutrition new:r/v ratio.
- Malnutrition prevention/prehab.
- MDT or nursing assistance with supportive care at time of risk screening (education, weekly weighs, intake monitoring, mealtime assistance).
- Meal support for pts with eating disorders by NAs.
- Medical malnutrition diagnosis.
- MST triaging and confirmation of risk.
- Multimedia standard educations.
- NA advising re d/c plan, e.g., discuss with GP.
- NA assistance post d/c scripting.
- NA HPHE education.
- NA pre assessment data.
- NAs doing d/c planning under guidelines.
- NAs or nurses for preference checks/flavours.
- NEMO therapeutic diet educations.
- Nurse records estimated intake.
- Nursing/AHA/interdisciplinary to commence SIMPLE malnutrition strategies on risk ax.
- Nursing/medical/AH malnutrition diagnoses.
- Nursing or DA blood monitoring/outpatients.
- Nutrition Assistant—rescreen, basic educations, assisted diagnosis (with DN sign off), e.g., on ward round.
- Overlearning repeat basic nutrition [messages].
- Patient centred care.
- Point of risk screen interdisciplinary education including discharge planning, e.g., talk to your GP.
- Power form diagnosis auto generates medical d/c summary 1 liner.
- Process for documentation and alternative care [to individualised DN care]/simple management.
- Process to support this [DNs stopping doing reviews where low value unless escalate nutrition support is requested].
- Pt reported experience measures.
- R/v referral + triage process.
- Re-referral criteria (ok to d/c) back to supportive.
- Reviewing MDT input/opportunities/low value meetings.
- Reviews or monitoring by non-dietitians.
- Room service.
- Seeing higher priority pts, e.g., using priority tool.
- Short/abbreviated assessments for at risk/malnourished (e.g., on mealtime monitoring).
- Short case for malnutrition.
- Short cases for malnutrition.
- Short malnutrition care WPI and/or cognitively impaired malnutrition pathway.
- SIMPLE audits.
- Standardised chart entry template, e.g., for new/review.
- Standardised protocols with templates with criteria.
- Systematised intake monitoring using CBORD [food service software] and NAs for intake monitoring.
- Systematised tube feeding/algorithms.
- Telehealth to [site removed for confidentiality] with assistant.
- Using TREND [workforce planning and workload management system], using DAs, etc, to streamline intake/prioritisation/reviews.
- Utilising procedures, protocols and pathways.

Acronyms used:

ADIME = Process of Assessment, Diagnosis, Intervention, and Monitoring/Evaluation
AH = Allied health
AHA = Allied health assistant
Ax = Assessment
d/c = Discharge
DA = Dietitian assistant
DN = Dietitian
GP = General practitioner
HENS = Home enteral nutrition support
HP = Health practitioner
HPHE = High protein high energy
IDT = Interdisciplinary team
MDT = Multi-disciplinary team
MST = Malnutrition screening tool
MST 2 = Malnutrition screening tool score of 2
NA = Nutrition Assistant
NEMO = Nutrition education materials online
PFM = Patient flow manager
PG-SGA = Patient generated subjective global assessment
Pt = Patient
r/v = Review
RACF = Residential aged care facility
SAM = Supplements as medicine
SGA = Subjective global assessment
SIMPLE = Systematised interdisciplinary malnutrition program for implementation and evaluation
WPI = Workplace instruction
